# Editorial: Immune transcriptome sequencing reveals secrets of veterinary infectious diseases

**DOI:** 10.3389/fvets.2023.1208088

**Published:** 2023-05-30

**Authors:** Mirinda van Kleef, Alri Pretorius

**Affiliations:** Agricultural Research Council – Onderstepoort Veterinary Research, Pretoria, South Africa

**Keywords:** transcriptomics, immunology, vaccine, control, pathogen, host, vector

An immune response to a disease-causing pathogen is complex. It involves multiple specialized cell types, modulating signals, gene and protein networks and functional pathways. Standard immunological assays only provide a rudimentary glimpse into this complex system. Immune transcriptome sequencing allows researchers to delve deeper and new bioinformatic tools assists in interpretation of differentially expressed genes and pathways. This provides a comprehensive functional biological extrapolation. Many pathogen causing diseases of veterinary importance do not have effective vaccines and control measures. Transcriptomics can provide insight into the host/pathogen/vector immune response interactions that can guide and assist disease control. It has also assisted in unraveling how a pathogen manipulates the host immune response resulting in immune evasion that may provide therapeutic targets for disease control (1, 2). Furthermore, transcriptomics can identify early disease specific immune response markers before symptoms begin, that will allow early and focused treatment thus preventing debilitating disease (3). Transcriptomics can also provide signatures of a protective immune response induced by a pathogen that will guide rational vaccine development (4, 5). The objective of this Research Topic was to further expand our immune knowledge base of veterinary diseases to assist in effective disease control.

*Theileria parva* transmitted by *Rhipicephalus appendiculatus* ticks to cattle, causes East Coast fever (ECF) in Africa. It has a complex life cycle involving sequential development in the tick and bovine host that complicates vaccine development. Atchou et al. are the first to report a study on the gene expression profile of the piroplasm stage (infective to tick vector) with reference to schizont stage (pathogenic form). This led to both increased gene expression knowledge of T. parva life cycle stages and several new candidate vaccine antigens identified. Combining these results with previous studies of the other life cycle stages, indicated that the majority genes are expressed throughout the life cycle but at various levels of expression. Furthermore, there are about 200 stage specific genes and those enriched in the piroplasm are indicated. The vaccine candidates were shown to be very immunogenic and they now need to be tested for their ability to induce protective immunity to ECF.

African swine fever (ASF) is a fatal disease of pigs and there is no vaccine available. Protection against ASF is only obtained after vaccination with attenuated strain and challenge with virulent homologous strain but not heterologous strain. Kholod et al. performed transcriptome analysis of peripheral blood mononuclear cells (PBMC) from attenuated ASFV strain immune animals and stimulated *in vitro* with virulent strains. The differentially expressed genes (DEGs) showed that in contrast to the homologous strain infection of the PBMC the heterologous strain resulted in an early immune response. This confirms that no recall or memory immune response was present to the heterologous strain explaining the lack of protection against heterologous challenge. DEGs between homologous and heterologous ASFV strains identified cytokine, chemokine and interferon-stimulated genes and genes involved in endocytoses and stress responses that may be important in ASFV heterologous strain cross protective immunity and vaccine development.

There are seven *Eimeria* species associated with the economically important disease of chicken coccidiosis. It is known that proteins derived from *Toxoplasma gondii* secretory organelles contribute to parasite invasion and survival. One such protein (EtHGRA9) of *Eimeria tenella*, homologous to *T. gondii* dense granule protein 9, is present in all life cycle stages of *E. tenella*. A transcriptome analysis study was undertaken to determine the role of this protein in the interaction between pathogen and its host (Wu et al.). Results indicate that the presence of EtHGRA9 protein has a significant role in several immune pathways and inflammatory responses (e.g., cytokine-cytokine and protein processing pathways). It is suggested that because this protein is involved in host/pathogen interaction it may therefore play an important role in control of this disease.

Foot-and-mouth disease virus (FMDV) is a highly contagious disease of cloven hooved animals. FMDV VP1 is a nucleocapsid protein known to be important in viral receptor binding, host cell entry and virus replication. In order to investigate in depth the role of FMDV VP1 in host pathogen interaction and viral replication, the signal pathways induced by this protein were explored in the study by Yang et al. Transcriptomic analysis of host cells transfected with plasmid expressed VP1 identified KEGG pathways mainly related to the immune system. The overexpression of certain chemokines as well as the down regulation of GBP1 transcripts promoted viral replication which was inhibited by the exogenous addition of recombinant GBP1 pDNA. Identifying the pathogenesis caused and the underlying mechanisms of immune pathways induced, provides a better understanding of the role of VP1 in FMDV infection and paves the way for future in depth research on host genes that confer resistance to FMDV infection.

In summary it is clear that transcriptome sequencing analysis revealed secrets regarding host pathogen interactions important for the development of effective control measures.

## Author contributions

All authors listed have made a substantial, direct, and intellectual contribution to the work and approved it for publication.
